# Supplementary material for: Impact of electronic health records on nursing workflow efficiency and predictive factors in Palestinian hospitals
Source: PLOS Digit Health. 2026 Mar 20;5(3):e0001318. doi: 10.1371/journal.pdig.0001318 (PMC13004382; doi:10.1371/journal.pdig.0001318)
Supplement: S1 File — The full data collection instrument used in the study, including all demographic items and workflow efficiency questions. (DOCX) [file pdig.0001318.s001.docx]

**Scientific Research Questionnaire**

**Section A: Sociodemographic & Work Environment Data:**

**Please answer the following questions by ticking (✔) or writing in the appropriate space**

- **Age: < 25 Years  25-35 Years  > 35 Years**
- **Gender: ** Male  Female
- **Years of experience in nursing: < 5 Years  5-10 Years  > 10 Years**
- **Have you received formal training on using the Electronic Medical Record system?**

 Yes  No

** How satisfied are you with the user-friendliness and ease of navigation of the Electronic Medical Record system?**

Strongly Disagree  Disagree  Neutral  Agree  Strongly Agree

- **How satisfied are you with the support you receive for using the Electronic Medical Record system in your workplace?**Strongly Disagree  Disagree  Neutral  Agree  Strongly Agree
- **How satisfied are you with the availability of computers and laptops to use the Electronic Medical Record system in your workplace?**

Strongly Disagree  Disagree  Neutral  Agree  Strongly Agree

**Section B: This section deals with workflow efficiency with Electronic Medical Records; Systems. Please mark (X) the response that best represents your situation:**

| **Item** | **Strongly Disagree**  **1** | **Disagree**  **2** | **Neutral**  **3** | **Agree**  **4** | **Strongly Agree**  **5** |
| --- | --- | --- | --- | --- | --- |
| **Access To Patient Information and time management** |  |  |  |  |  |
| 1. The Electronic Medical Record system allows me to access and retrieve patient records **quickly and easily** than with the paper documentation. |  |  |  |  |  |
| 2. The Electronic Medical Record system has reduced the time I spend on documentation tasks compared to paper documentation. |  |  |  |  |  |
| **Interruptions and Distractions** |  |  |  |  |  |
| 3.  I experience fewer interruptions in my workflow when using the Electronic Medical Record system compared to paper documentation. |  |  |  |  |  |
| 4. Technical issues (e.g., system crashes, slow response) frequently delay my documentation tasks when using the Electronic Medical Record system. |  |  |  |  |  |
| **Coordination of Care** |  |  |  |  |  |
| 5. The Electronic Medical Record system improves teamwork and coordination in patient care compared to paper documentation. |  |  |  |  |  |
| 6. The Electronic Medical Record system facilitates faster and clearer communication of patient updates compared to paper documentation. |  |  |  |  |  |
| **Tracking Patient Progress** |  |  |  |  |  |
| 7. I find it easier to track patient progress and outcomes using the Electronic Medical Record system compared to paper documentation. |  |  |  |  |  |
| 8. The Electronic Medical Record system provides timely alerts about changes in patient condition. |  |  |  |  |  |
| **Accuracy of Documentation** |  |  |  |  |  |
| 9.The Electronic Medical Record system helps me complete documentation more accurately compared to paper documentation. |  |  |  |  |  |

**End of the Questionnaire
Thank You**
