# Supplementary material for: Impact of electronic health records on nursing workflow efficiency and predictive factors in Palestinian hospitals
Source: PLOS Digit Health. 2026 Mar 20;5(3):e0001318. doi: 10.1371/journal.pdig.0001318 (PMC13004382; doi:10.1371/journal.pdig.0001318)
Supplement: S2 File — A completed STROBE (Strengthening the Reporting of Observational Studies in Epidemiology) checklist detailing adherence to reporting guidelines for cross-sectional studies. (DOCX) [file pdig.0001318.s002.docx]

**STROBE Checklist**

Title: **The Impact of Electronic Health Records on Nursing Workflow Efficiency and Predictive Factors in Palestinian Governmental Hospitals**

| **Item No** | **Recommendation** | **Reported on Page #** |
| --- | --- | --- |
| **Title and abstract** |  |  |
| 1(a) | Indicate the study’s design with a commonly used term in the title or the abstract | 1 (Abstract: “A quantitative, cross-sectional study design was employed”) |
| 1(b) | Provide in the abstract an informative and balanced summary of what was done and what was found | 1 (Abstract: Summarizes background, methods, results, and conclusions, including aim, sample, data collection, key findings, and implications) |
| **Introduction** |  |  |
| 2 | Explain the scientific background and rationale for the investigation being reported | 2–4 (Introduction: Discusses EMRs’ role in healthcare, global and regional context, challenges in Palestine, and knowledge gap) |
| 3 | State specific objectives, including any prespecified hypotheses | 4 (Introduction: States aim to assess EMR influence on workflow efficiency and contributing factors among nurses in Palestinian governmental hospitals) |
| **Methods** |  |  |
| 4 | Present key elements of study design early in the paper | 5 (Methods - Design: “This study employed a quantitative, cross-sectional design”) |
| 5 | Describe the setting, locations, and relevant dates, including periods of recruitment, exposure, follow-up, and data collection | 6–7 (Methods - Setting: Six governmental hospitals in West Bank; Data Collection: February–March 2025) |
| 6(a) | Give the eligibility criteria, and the sources and methods of selection of participants | 6 (Methods - Population, Sample and Sampling, Inclusion and Exclusion Criteria: Nurses with ≥3 months EMR experience, prior paper-based system experience; excludes mixed documentation units and non-clinical staff) |
| 7 | Clearly define all outcomes, exposures, predictors, potential confounders, and effect modifiers. Give diagnostic criteria, if applicable | 6–7 (Methods - Instrumentation: Defines outcome as workflow efficiency via 9-item Likert scale; predictors include user-friendliness, training, technical support, age, gender, experience, shifts, computer availability; no diagnostic criteria) |
| 8 | For each variable of interest, give sources of data and details of methods of assessment (measurement). Describe comparability of assessment methods if there is more than one group | 6–7 (Methods - Instrumentation: Structured questionnaire, 5-point Likert scale for efficiency; demographic and environmental variables; content validity and pilot study described; single group, no comparability needed) |
| 9 | Describe any efforts to address potential sources of bias | 7 (Methods - Instrumentation: Pilot study with 25 nurses for clarity; content validity via expert review; Cronbach’s α=0.88; excluded pilot participants to avoid bias) |
| 10 | Explain how the study size was arrived at | 6 (Methods - Sample and Sampling: G*Power for regression (n=160, f²=0.15, α=0.05, 95% power); Raosoft calculator (n=184, 95% CI, 5% margin); increased to 210 for dropouts) |
| 11 | Explain how quantitative variables were handled in the analyses. If applicable, describe which groupings were chosen and why | 7 (Methods - Data Analysis: Descriptive statistics, multiple linear regression; Likert scale categorized into 5 levels per Pimentel (2019) for equitable intervals) |
| 12(a) | Describe all statistical methods, including those used to control for confounding | 7 (Methods - Data Analysis: SPSS v29; descriptive statistics (frequencies, means, SD); multiple linear regression for predictors; confounders like experience years included) |
| 12(b) | Describe any methods used to examine subgroups and interactions | Not applicable (No subgroup or interaction analyses reported) |
| 12(c) | Explain how missing data were addressed | Not explicitly reported (190/210 questionnaires completed; assumed minimal missing data due to high response rate of 90.4%) |
| 12(d) | If applicable, describe analytical methods taking account of sampling strategy | 6–7 (Methods - Sample and Sampling: Convenience sampling; regression analysis accounts for non-probability sampling limitations) |
| 12(e) | Describe any sensitivity analyses | Not applicable (No sensitivity analyses reported) |
| **Results** |  |  |
| 13(a) | Report numbers of individuals at each stage of study—e.g., numbers potentially eligible, examined for eligibility, confirmed eligible, included in the study, completing follow-up, and analysed | 7 (Methods - Data Collection: ~350 nurses eligible; 210 questionnaires distributed; 190 completed and analyzed, 90.4% response rate) |
| 13(b) | Give reasons for non-participation at each stage | Not explicitly reported (Assumed due to incomplete or unreturned questionnaires, as 20/210 not returned) |
| 13(c) | Consider use of a flow diagram | Not applicable (No flow diagram included; simple recruitment process described) |
| 14(a) | Give characteristics of study participants (e.g., demographic, clinical, social) and information on exposures and potential confounders | 8 (Results - Table 1: Age, gender, shift, training; Table 2: Environmental factors) |
| 14(b) | Indicate number of participants with missing data for each variable of interest | Not explicitly reported (Assumed no missing data in analyzed sample of 190) |
| 15 | Report numbers of outcome events or summary measures | 8–10 (Results - Tables 3–4: Workflow efficiency means, SD, percentages; M=3.59) |
| 16(a) | Give unadjusted estimates and, if applicable, confounder-adjusted estimates and their precision (e.g., 95% confidence interval). Make clear which confounders were adjusted for and why they were included | 10 (Results - Table 5: Regression β, SE, t, 95% CI, p-values for predictors) |
| 16(b) | Report category boundaries when continuous variables were categorized | 8 (Results - Table 4: Likert mean cutoffs: 1.00–1.79 very low, etc.) |
| 16(c) | If relevant, consider translating estimates of relative risk into absolute risk for a meaningful time period | Not applicable (No risk estimates; regression coefficients reported) |
| 17 | Report other analyses done—e.g., analyses of subgroups and interactions, and sensitivity analyses | 10 (Results - Table 5: Multiple regression as primary; no additional subgroups) |
| **Discussion** |  |  |
| 18 | Summarise key results with reference to study objectives | 11–12 (Discussion: 70% high efficiency; predictors: user-friendliness, training, support, age) |
| 19 | Discuss limitations of the study, taking into account sources of potential bias or imprecision. Discuss both direction and magnitude of any potential bias | 13 (Strengths and Limitations: Cross-sectional limits causality; convenience sampling bias; self-report bias) |
| 20 | Give a cautious overall interpretation of results considering objectives, limitations, multiplicity of analyses, results from similar studies, and other relevant evidence | 11–13 (Discussion: Aligns with global studies; cautious due to context-specific challenges) |
| 21 | Discuss the generalisability (external validity) of the study results | 13 (Strengths and Limitations: Limited to governmental hospitals in West Bank; suggests future private hospital studies) |
| **Other information** |  |  |
| 22 | Give the source of funding and the role of the funders for the present study and, if applicable, for the original study on which the present article is based | Not reported (No funding sources mentioned in manuscript) |
