# Supplementary material for: Impact of electronic health records on nursing workflow efficiency and predictive factors in Palestinian hospitals
Source: PLOS Digit Health. 2026 Mar 20;5(3):e0001318. doi: 10.1371/journal.pdig.0001318 (PMC13004382; doi:10.1371/journal.pdig.0001318)
Supplement: Attachment — Submitted filename: Paper Review .pdf [file pdig.0001318.s003.pdf]

Dear Authors,

Thank you for submitting your important work on the impact of EHRs on nursing workflow in Palestinian governmental hospitals. This is a very valuable and timely study, especially given the unique resource-constrained environment you detail. Your finding that usability, training, and technical support are key predictors of efficiency is a crucial contribution to the global discussion on health technologies in low-resource settings.

To ensure your manuscript meets the high standards, here are some constructive recommendations.

- Your study is a quantitative cross-sectional design. To adhere to international reporting standards, please ensure you state compliance with the STROBE guidelines (Strengthening the Reporting of Observational Studies in Epidemiology) and include the completed STROBE checklist as a supplementary file.

- I noticed a discrepancy in how the Age variable is reported. The Abstract suggests a positive effect (β=0.223), but the regression table (Table 4) shows a negative standardized coefficient (β= -0.223). Since the discussion confirms that younger nurses (lower age) are associated with higher efficiency, the negative sign in Table 4 is statistically correct for the *Age* variable. Please ensure the Abstract and Discussion consistently reference either the Age variable (with the negative sign) or the 'Younger Nurses' concept (with the positive implication) to avoid confusing the reader.

- You confirmed that the VIF assumption was met (VIF < 3.0). For added rigor, consider briefly reporting the range of VIF values in the results section or in a note beneath Table 4.

- Your study's most distinctive finding is the high rate of interruptions due to technical issues (50.8% of nurses) rooted in infrastructure (power outages, instability). This is a stronger barrier than the 'system complexity' often reported in developed countries. I suggest dedicating a specific paragraph to elaborating on *how* these real-life infrastructure challenges uniquely constrain EHR benefits in Palestine, significantly differentiating your work.

- Briefly comment on why demographic factors like Gender (p=0.982) and Years of Experience (p=0.250) were *not* significant predictors. This shows a complete analysis of the tested variables.

Addressing these points will significantly strengthen your manuscript, ensuring its findings are fully accessible and its recommendations are clear and implementable.
Best regards!
