# Supplementary material for: Impact of electronic health records on nursing workflow efficiency and predictive factors in Palestinian hospitals
Source: PLOS Digit Health. 2026 Mar 20;5(3):e0001318. doi: 10.1371/journal.pdig.0001318 (PMC13004382; doi:10.1371/journal.pdig.0001318)
Supplement: Attachment — Submitted filename: Reponse to The Reviwers.docx [file pdig.0001318.s005.docx]

## ****Editor’s Comment****

**Journal Requirements 1**

Please ensure that your Ethics Statement is available in its entirety at the beginning of your Methods section, under a subheading “Ethics Statement”.

## ****Author Response****

Thank you for the comment. The full Ethics Statement has now been placed at the very beginning of the Methods section under the required subheading “Ethics Statement.”

**Journal Requirements 2**

We have noticed that you have uploaded Supporting Information files, but you have not included a list of legends. Please add a full list of legends for your Supporting Information files after the references list.

## ****Author Response****

Thank you for pointing this out. A complete “List of Supporting Information Legends” has now been added after the References section. The list includes legends for all supplementary files submitted with the manuscript, including the STROBE checklist, the data collection sheet, and the ethical approval letter.

**Journal Requirements 3**

*PLOS requires that all data underlying the findings be freely available in a public repository, within the manuscript, or as supplementary files, unless public deposition would violate ethical or legal restrictions. If the data cannot be made fully public due to privacy concerns, please explain this so an exemption can be reviewed.*

## ****Author Response****

Thank you for this clarification. We remain fully **open to sharing the dataset**, and it will be provided **to any qualified researcher upon reasonable request**, following standard research regulations. A revised Data Availability Statement has been added to the submission system “The dataset is de-identified and can be shared with researchers upon reasonable request"

**Point by Point Response to Reviewer Comments**

**Reviewer 1 Comment 1:**
*There are inconsistencies between the abstract, results text, and Table 4. For example, age is reported as a significant predictor with β = –0.223 in Table 4, but the abstract states “younger nurses (β = 0.223)” and the discussion describes a “moderate positive correlation.”*

**Response:**
Thank you for highlighting this important inconsistency. We have carefully reviewed the regression output and confirm that the standardized regression coefficient for Age is negative (β = –0.223, p = 0.037), indicating that lower age (i.e., younger nurses) is associated with higher workflow efficiency. The positive sign reported in the abstract and the phrasing of “moderate positive correlation” in the discussion were errors in interpretation.

To resolve this issue, we have:

1. Corrected the Abstract to consistently reflect the negative standardized beta coefficient.
2. Revised the Results section to maintain consistent reporting with Table 4.
3. Rewritten the relevant paragraph in the Discussion to accurately interpret the negative coefficient in terms of younger age predicting higher workflow efficiency.

**Reviewer 1 Comment 2:**
*Confidence intervals and p-values look inconsistent (e.g., some CIs do not include 0 but p-values are non-significant, and vice versa). Please re-run the regression, clearly specify whether coefficients are standardized or unstandardized, and correct the table, abstract, and discussion accordingly.*

**Response:**
We thank the reviewer for this important observation. The initial regression table contained formatting and labeling errors in the 95% confidence interval columns, which led to apparent inconsistencies between confidence intervals and p-values. We have now re-ran the multiple linear regression analysis, carefully verified all outputs, and ensured full consistency between the confidence intervals, p-values, and standardized regression coefficients.

We also clarified that all regression coefficients reported in Table 4 are standardized beta coefficients. The regression table has been fully corrected, and all corresponding values in the Abstract, Results, and Discussion sections have been revised accordingly to ensure complete statistical consistency.

**Reviewer 1 Comment 3:**
*The second section of the questionnaire (9 items, 4 domains) is only briefly described. Please provide more detail about exact items (or a clear pointer to the full instrument in Supplementary File 1), response options, scoring approach, handling of reverse-coded items, and how the global “workflow efficiency” score entered the regression. Please clarify whether the tool was developed in English or translated.*

**Response:**
We thank the reviewer for this important methodological suggestion. We have expanded the description of the second section of the questionnaire in the Methods (Instrumentation section) to provide full clarity regarding: (1) the exact number of items and domains, (2) response options, (3) scoring procedure, (4) handling of reverse-coded items, (5) calculation of the global workflow efficiency score, and (6) how this score was used as the dependent variable in the regression model.

We also explicitly clarified that the instrument was developed in English, reviewed by experts, pilot-tested, and that the complete version of the questionnaire is available in Supplementary File 1. These revisions improve the transparency and reproducibility of the study.

**Reviewer 1 Comment 4:**
*The study uses convenience sampling from governmental hospitals only. The limitations section mentions some biases, but this needs to be more explicit: selection bias, potential over-representation of nurses more comfortable with technology, and limited generalizability to private or rural facilities.*

**Response:**
We thank the reviewer for this important methodological observation. The Limitations section has been revised to explicitly acknowledge the use of convenience sampling, the resulting potential for selection bias, possible over-representation of nurses who are more comfortable with technology, and the restricted generalizability of the findings to governmental hospitals only. We have also clarified that the results may not be fully generalizable to private or rural healthcare settings. These revisions enhance the transparency and rigor of the study.

**Reviewer 1 Comment 5:**
*“The manuscript does not adequately describe the EHR system(s) in use (vendor or local system, core modules, years since implementation, whether nursing workflows are fully digital).”*

**Response:**

1. **Thank you for the comment.** We agree that additional detail about the EHR system is necessary for clarity and reproducibility.
2. **System name added.** We have now specified that all governmental hospitals in Palestine use **Avicena**, the nationally implemented EHR platform adopted by the Palestinian Ministry of Health.
3. **Local development clarified.** We added that Avicena is a locally developed system tailored to governmental hospital needs.
4. **Modules described.** We included a concise summary of the core modules accessible to nurses (nursing documentation, vital signs, medication administration, orders/results, progress notes, etc.).
5. **Implementation timeline added.** We indicated the years of rollout (2018–2020), noting that all sampled hospitals have been fully onboard for at least 3 years.
6. **Workflow digitization clarified.** We added that nursing workflows in governmental hospitals are predominantly fully digital, with limited paper fallback procedures during outages.
7. **Manuscript updated.** A new paragraph has been added to the Methods → Design and Setting section

**Reviewer 2**

**Reviewer 2 Comment 1:**
“PLOS journals typically require deposition of a minimal de-identified dataset in a public repository unless there is a clear, justified ethical barrier. Please revise the Data Availability statement to explicitly align with journal policy.”

### ****Response:**** We appreciate the reviewer’s guidance on data-sharing requirements., We confirm that the minimal de-identified dataset can be shared. We updated the Data Availability section to state that the dataset supporting the findings is available from the corresponding author upon reasonable request.

**Reviewer 2 Comment 2:**
*Your study is a quantitative cross-sectional design. To adhere to international reporting standards, please ensure you state compliance with the STROBE guidelines (Strengthening the Reporting of Observational Studies in Epidemiology) and include the completed STROBE checklist as a supplementary file.*

Response:
Thank you for this valuable comment. We confirm that this study follows the STROBE (Strengthening the Reporting of Observational Studies in Epidemiology) guidelines for reporting cross-sectional studies. Accordingly, a statement indicating STROBE compliance has now been added to the Methods section of the manuscript. In addition, the completed STROBE checklist has been prepared and submitted as a supplementary file to ensure full transparency and adherence to international reporting standards.

**Reviewer 2 Comment 3:**

*I noticed a discrepancy in how the Age variable is reported. The Abstract suggests a positive effect (beta=0.223), but the regression table (Table 4) shows a negative standardized coefficient (beta= -0.223). Since the discussion confirms that younger nurses (lower age) are associated with higher efficiency, the negative sign in Table 4 is statistically correct for the Age variable. Please ensure the Abstract and Discussion consistently reference either the Age variable (with the negative sign) or the 'Younger Nurses' concept (with the positive implication) to avoid confusing the reader.*

**Response:**
Thank you for highlighting this important inconsistency. We have carefully reviewed the regression output and confirm that the standardized regression coefficient for Age is negative (β = –0.223, p = 0.037), indicating that lower age (i.e., younger nurses) is associated with higher workflow efficiency. The positive sign reported in the abstract and the phrasing of “moderate positive correlation” in the discussion were errors in interpretation.

**Reviewer 2 Comment 4:**
*You confirmed that the VIF assumption was met (VIF < 3.0). For added rigor, consider briefly reporting the range of VIF values in the results section or in a note beneath Table 4.*

**Response:**
Thank you for this important suggestion. To enhance the transparency and rigor of the regression analysis, we have now explicitly reported the range of the Variance Inflation Factor (VIF) values. The VIF range (1.12–2.41) has been added as a diagnostic statement in the Results section

**Reviewer 2 Comment 5:**
*Your study's most distinctive finding is the high rate of interruptions due to technical issues (50.8% of nurses) rooted in infrastructure (power outages, instability). This is a stronger barrier than the 'system complexity' often reported in developed countries. I suggest dedicating a specific paragraph to elaborating on how these real-life infrastructure challenges uniquely constrain EHR benefits in Palestine, significantly differentiating your work.*

**Response:**
We sincerely thank the reviewer for highlighting this important and distinctive contribution of our study. In response, we have added a new dedicated paragraph in the Discussion section that specifically elaborates on how real-life infrastructure challenges, including power outages, unstable electricity, and limited technical infrastructure, uniquely constrain the benefits of EHR implementation in Palestine. This paragraph contrasts our findings with those from developed countries where system complexity is the dominant barrier; thereby further strengthening the contextual and scientific novelty of our work.

**Reviewer 2 Comment 6:**
*Briefly comment on why demographic factors like Gender (p = 0.982) and Years of Experience (p = 0.250) were not significant predictors. This shows a complete analysis of the tested variables.*

**Response:**
Thank you for this insightful suggestion. In response, we have added a brief explanatory paragraph in the Discussion section addressing the non-significant effects of gender and years of experience on workflow efficiency.
