# Supplementary material for: Impact of electronic health records on nursing workflow efficiency and predictive factors in Palestinian hospitals
Source: PLOS Digit Health. 2026 Mar 20;5(3):e0001318. doi: 10.1371/journal.pdig.0001318 (PMC13004382; doi:10.1371/journal.pdig.0001318)
Supplement: Attachment — Submitted filename: Response to the reviwers.docx [file pdig.0001318.s006.docx]

**Point by Point Response to Reviewer 1**

**Reviewer Comment 1**

*“Please explicitly state how each predictor was coded in the regression (e.g., age and experience as continuous years vs. grouped categories; gender coding; Likert predictors treated as continuous). This is essential for interpretation of the unstandardized coefficients (B) and CIs.”*

**Author Response:**
T Thank you for this comment. We have clarified how all predictors were coded in the multiple linear regression model. Age and years of experience were entered as continuous variables (years); gender and EHR training were binary-coded; and Likert-scale predictors (user-friendliness, IT support, access to computers) were treated as continuous variables. This information has been added to the Data Analysis section to aid interpretation of unstandardized coefficients and confidence intervals.

**Reviewer Comment 2**

*Please ensure the manuscript uses one consistent framing, either “Age (negative β)” or “Younger nurses (positive implication)”, but not both. Also confirm the Discussion does not describe a “positive correlation” if the standardized beta for age is negative.*

**Author Response:**

Thank you for this comment. We revised the manuscript to use a single, consistent framing when interpreting the age variable. Age is now consistently described as having a negative standardized beta, indicating that increasing age is associated with lower perceived workflow efficiency. We also removed any wording implying a “positive correlation” and aligned the Discussion with the direction of the regression coefficient.

**Reviewer Comment 3**

*“The 9-item workflow efficiency instrument has good internal consistency, but please add a brief justification for using a single global mean score. If feasible, add a short factor-structure check (even exploratory) or a rationale for not doing so.”*

**Author Response:**
Thank you for this comment. We have added a brief justification for using a single global mean workflow efficiency score. Although the instrument includes items covering related aspects of workflow, it was designed and applied to measure overall workflow efficiency as a unidimensional construct. Given the high internal consistency (Cronbach’s α = 0.88) and the study’s focus on overall efficiency rather than subdomain interpretation, a single composite mean score was used. We have also added a rationale explaining why a factor-structure analysis was not conducted.

### ****Reviewer comment 4****

*Because both predictors (e.g., perceived usability/support) and outcomes are self-reported in the same survey, please acknowledge potential common-method variance and that “workflow efficiency” is perceived rather than objectively measured (e.g., no time-motion or EHR log data).*

**Author Response:**
We thank the reviewer for this important observation. We have revised the manuscript to explicitly acknowledge the potential for common-method variance due to the use of a single self-reported questionnaire for both predictors and outcomes. We have also clarified that workflow efficiency was assessed as a perceived construct rather than through objective measures such as time-motion studies or EHR log data. These limitations have now been explicitly stated in the Strengths and Limitations section.

### ****Reviewer comment 5****

*Double-check percentages in Table 1 (some values appear inconsistent with n).*

**Author Response:**
Thank you for highlighting this issue. We carefully rechecked all frequencies and percentages in Table 1 and corrected minor calculation errors. Specifically, the percentage for nurses who did not receive EHR training has been revised to accurately reflect the sample size. All percentages in Table 1 are now consistent with *n = 185*.

**Reviewer comment 6**

*Make sure the reverse-coded technical-interruptions item still reports its mean/SD (or clarify why it is omitted).*

**Author Response:**
Thank you for this comment. We have revised Table 2 to report the mean and standard deviation for the reverse-coded technical-interruptions item for transparency. The item is clearly marked as reverse-coded, and its mean/SD are now presented consistently with the other workflow efficiency items.

**Reviewer comment 7**

*A few grammatical issues remain.*

**Author Response:**
Thank you for noting this. We conducted a thorough grammatical and language review of the manuscript and corrected minor grammatical errors, punctuation issues, and verb-tense inconsistencies throughout the text. These revisions did not alter the meaning or interpretation of the findings.
